# Pure Graphene Oxide Vertical p–n Junction with Remarkable Rectification Effect

**DOI:** 10.3390/molecules26226849

**Published:** 2021-11-13

**Authors:** Yan Fan, Tao Wang, Yinwei Qiu, Yinli Yang, Qiubo Pan, Jun Zheng, Songwei Zeng, Wei Liu, Gang Lou, Liang Chen

**Affiliations:** 1 Department of Optical Engineering, School of Information and Industry, Zhejiang A&F University, Hangzhou 311300, China; 20060068@zafu.edu.cn (Y.F.); 18435111940@163.com (T.W.); qyw_981012@163.com (Y.Q.); yangyinli258@163.com (Y.Y.); p1482565341@163.com (Q.P.); zhengjun@zafu.edu.cn (J.Z.); zsw@zafu.edu.cn (S.Z.); 2 College of Physics and Electronic Information Engineering, Zhejiang Normal University, Jinhua 321004, China

**Keywords:** undoped p–n junction, vertical p–n junction, graphene oxide

## Abstract

Graphene p-n junctions have important applications in the fields of optical interconnection and low–power integrated circuits. Most current research is based on the lateral p-n junction prepared by chemical doping and other methods. Here, we report a new type of pure graphene oxide (pGO) vertical p-n junctions which do not dope any other elements but only controls the oxygen content of GO. The I–V curve of the pGO vertical p–n junction demonstrates a remarkable rectification effect. In addition, the pGO vertical p–n junction shows stability of its rectification characteristic over long-term storage for six months when sealed and stored in a PE bag. Moreover, the pGO vertical p–n junctions have obvious photoelectric response and various rectification effects with different thicknesses and an oxygen content of GO, humidity, and temperature. Hall effect test results show that rGO is an n–type semiconductor; theoretical calculations and research show that GO is generally a p–type semiconductor with a bandgap, thereby forming a p–n junction. Our work provides a method for preparing undoped GO vertical p–n junctions with advantages such as simplicity, convenience, and large–scale industrial preparation. Our work demonstrates great potential for application in electronics and highly sensitive sensors.

## 1. Introduction

As graphene (an atomically thin hexagonal lattice) has electrical characteristics such as high mobility of charge carriers [1,2] and high conductivity [2,3], while the p–n junction is the basic composition of various electronic devices, graphene-based p-n junctions have shown potential for a variety of applications, including low–power integrated circuits [4,5], energy conversion and storage [6,7], sensors [8,9], optical devices [10,11,12], solid state solar cells [13,14], and other fields. Both graphene p-n junctions and heterojunctions have demonstrated the rectification effect [15,16]. Recently, we have reported the direct observation of Na–Cl crystals/Graphene heterojunction [17] and Ca–Cl crystals/Graphene heterojunction [18] with rectification effect in rGO films. However, these graphene p–n junctions and heterojunctions reported are fabricated via chemical doping [19,20,21] or electrostatic doping [22,23,24], and most of them are lateral p–n junction [25,26,27] and heterojunctions [17,18]. Chemical doping is a method wherein nitrogen [28], potassium [29], and other elements are doped into graphene to control the semiconductor type of graphene. Electrostatic doping uses electric field effects to control the carrier type and concentration of graphene. However, both electrostatic doping and chemical doping have many drawbacks, such as substrate defects formed by high voltage [28]; they require complicated technologies for doping and a specific electronical or chemical setup [19]. It is very complicated and tedious to fabricate graphene p–n junctions via the doping method. Therefore, these traditional doping methods for p–n junctions greatly limit the practical application of graphene p–n junctions in electronics and sensors. Moreover, the lateral graphene p–n junction shows a limited rectification effect [25,26,27] due to the Klein tunnel effect [30], in which the transmission probability for a single square barrier could reach 100% in a certain range of energies [31]. In addition, the area of the depletion layer depends on the contact area between the GO and rGO films, which can be large-scale preparation for vertical structure, superior to lateral ones. Thus, a vertical graphene oxide p–n junction is reported [16], which shows better rectification characteristics than lateral p–n junctions, since it is not affected by Klein tunnel effect [32]. Although the results of the rectification effect and photoresponsivity are demonstrated in [16], further study of vertical graphene oxide p–n junction is needed for practical applications.

In this work, we fabricated a vertical p–n junction of pure GO (pGO) films by changing the oxygen content of GO, which does not require any doping process. X–ray photoelectron spectrometer spectra (XPS) results showed that there are no observable impurity elements signals, demonstrating that the pGO p–n junctions are undoped. Meanwhile, this pGO p–n junction has a remarkable rectification effect, demonstrating superior rectification performance compared to those lateral p–n junctions [26] and vertical GO p–n junctions reported previously [16]. In addition, this p–n junction shows stability of rectification characteristic over long–term storage for six months with sealed and stored in a PE bag. Moreover, the I–V curve of pGO vertical junctions showed varies rectification effects with thicknesses and oxygen content of GO, and that is sensitive to humidity and temperature. This method demonstrates advantages such as simplicity, convenience, and makes pGO p–n junctions have great potential in the field of sensors.

## 2. Experimental Section

### 2.1. Materials

Graphite powder (325 mesh), sulphuric acid (H_2_SO_4_), potassium persulfate (K_2_S_2_O_8_), phosphorus pentoxide (P_2_O_5_), potassium permanganate (KMnO_4_), hydrogen peroxide (H_2_O_2_), and hydrochloric acid (HCl) were used in this study. All chemical regents were purchased from shanghai Aladdin Biochemical Technology Co., Ltd. (6th Floor, Sanda Building, No.196 Xinjinqiao Road, Pudong New Area, Shanghai), and used with analytically pure deionized (DI) water with 18.2 MΩ·cm^−1^ in all experiments.

### 2.2. Fabrication of GO Suspension

GO suspension (5 mg/mL, 1 mL) was prepared from graphite powder according to the modified hummers method as previously reported [33,34]. Graphite powder was pre-oxidized by concentrated H_2_SO_4_, K_2_S_2_O_8_, and P_2_O_5_ solution and stirred continuously for 4.5 h. The mixture was then centrifuged and washed with deionized (DI) water. After vacuum drying, pre–oxidized graphite was obtained. This was further oxidized in concentrated H_2_SO_4_ and KMnO_4_, diluted with DI water, and then added with 30% H_2_O_2_. The product was centrifuged and washed with a 1:10 HCl solution and DI water sequentially to remove impurities. Finally, the GO suspension with a concentration of about 5 mg/mL was prepared.

### 2.3. Composite Film Prepared by Two-Step Drop-Coating Method

The preparation of GO films. The GO films were prepared by drop–casting the GO suspension (5 mg/mL, 1 mL) droplets onto a smooth paper substrate [34,35], drying thoroughly at 70 °C for 12 h under ambient conditions. After that, they were peeled off, rinsed and soaked with DI water for more than half hour to remove potential impurities, then dried at 70 °C for 12 h. These prepared GO films were used for further preparation of a series of pGO vertical p–n junctions.

The preparation of pGO vertical p–n junctions. The prepared GO films were further reduced at 180 °C to obtain rGO films under ambient conditions. Then, drop–casting another 1 mL GO suspension on the rGO films, followed with drying at 70 °C for 12 h. Next, the prepared rGO–GO films were further reduced at 180 °C for 2 h to obtain pGO vertical p-n junctions. For GO–GO vertical composite films, drop-casting another 1 mL GO suspension on the GO films, followed with drying at 70 °C for 12 h.

For pGO vertical p-n junctions with different oxygen content of GO, the prepared rGO–GO films were reduced with a corresponding temperature range of 70 °C to 180 °C.

For pGO vertical p–n junctions with different thicknesses of GO, drop–casting 0.2~1 mL GO suspension on the rGO films was carried out, followed with drying at 70 °C for 12 h. Next, the prepared rGO–GO films were further reduced at 180 °C for 2 h, to obtain rGO–rGO vertical composite films with different thickness.

For the ultrathin pGO vertical p–n junctions, a specific method involves the GO suspension being heated at 70 °C for 40 min in order to obtain an ultrathin and transparent GO film on the suspension. Then, the ultrathin GO film was transferred to the surface of a rGO film. Finally, the composite film was dried at 70 °C for 12 h.

All of the prepared films were rinsed and soaked with DI water for more than half hour to remove potential impurities.

### 2.4. Instrument for Characterization and Measurement

The topographic images and height profiles of pGO vertical p–n junctions were measured by scanning electron microscope (TM4000Plus, Hitachi, Tokyo, Japan). X–ray photoelectron spectrometer spectra (XPS, Scientific compass, Hangzhou, China) results of pGO vertical p–n junctions were characterized by Thermo Scientific K–Alpha (Thermo, Waltham, MA, USA). The I–V curves of pGO vertical p–n junctions were measured by KEITHLEY 2611B (Keithley, Cleveland, OH, USA) with the resistance of about 0.2 Ω. The resistivity of rGO films and GO films were measured by four–probe Vanderbilt method at room temperature. 

## 3. Results and Discussion

### 3.1. Preparation and Characterization

**Figure 1:**
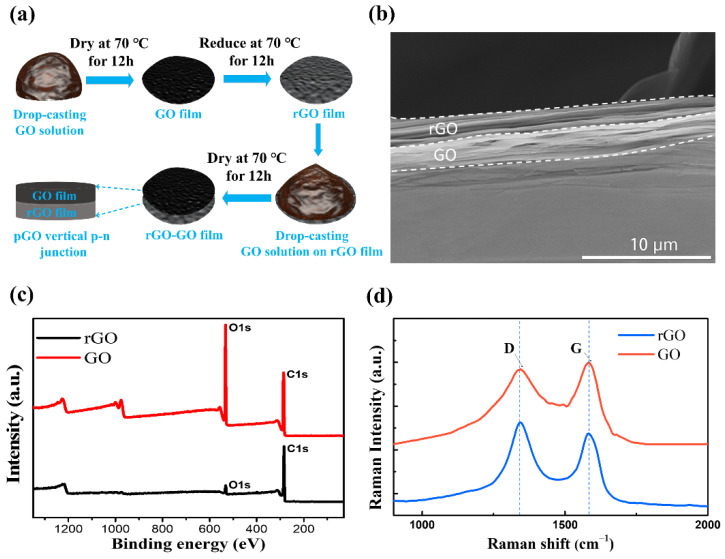
(**a**) A schematic of the preparation process of pGO vertical p–n junctions. (**b**) Cross–sectional scanning electron microscope (SEM) image of pGO vertical p–n junctions. (**c**) X–ray photoelectron spectrometer spectra (XPS) of GO and rGO film. (**d**) Raman spectra of GO and rGO films.

As illustrated in Figure 1a, the pGO vertical p–n junction was composed of rGO–GO vertical composite films, which were prepared with GO suspension by a two–step drop–coating method (for details please see the Methods section). Due to the different drying temperatures of 70 °C for GO and 180 °C for rGO in the composite structure, the electrical properties of the GO and rGO films are different, forming a potential p–n junction. Scanning electron microscope (SEM) images showed stacked sheets of pGO vertical p–n junctions, while the rGO film has more compacted structures than that of GO film, as shown in Figure 1b. The potential p–n junction is composed of two different materials, namely an rGO film and a GO film.

We used XPS to detect the atomic concentrations and oxygen–containing group distribution. Figure 1c shows the survey XPS scans of the GO and rGO films. The data were calibrated by refereeing to a binding energy of 284.6 eV, belonging to carbon to compensate for the surface charge effect. The XPS spectra of all samples reveal two peaks at 284.6 and 534.0 eV, corresponding to the C1s and O1s core levels, respectively. For GO film, the content of carbon atoms and oxygen atoms is 73.35% and 26.65%, respectively. For rGO film, the content of carbon and oxygen atoms in rGO is 93.78% and 6.22%, respectively. Notably, rGO’s oxygen content decreased from 26.65% to 6.22% compared to GO, while its carbon content increased from 73.35% to 93.78%, suggesting that the electrical properties of GO can be facilely varied with temperature. Importantly, we can see that there are no observable impurity elements signals, proving that the p-n junction is non–doped.

We further measured the defects of GO and rGO films by Raman spectrum. As shown in Figure 1d, two major peaks (located at 1340 cm^–1^ and 1580 cm^–1^) are observed in the Raman spectra, which are denoted as D and G bands, respectively. The intensity ratio of I_(D)_/I_(G)_ of GO film and rGO film is 0.98 and 1.06, indicating the similar defects both in GO and rGO films [36]. The slight increase in I_(D)_/I_(G)_ of rGO compare to GO is attributed to the increased defects during the reduction at 180 °C, in which the oxygen–containing groups such as hydroxyl and carboxyl groups have been removed. It was consistent with the XPS results in Figure 1c.

### 3.2. Measurement of Rectification Performance

**Figure 2:**
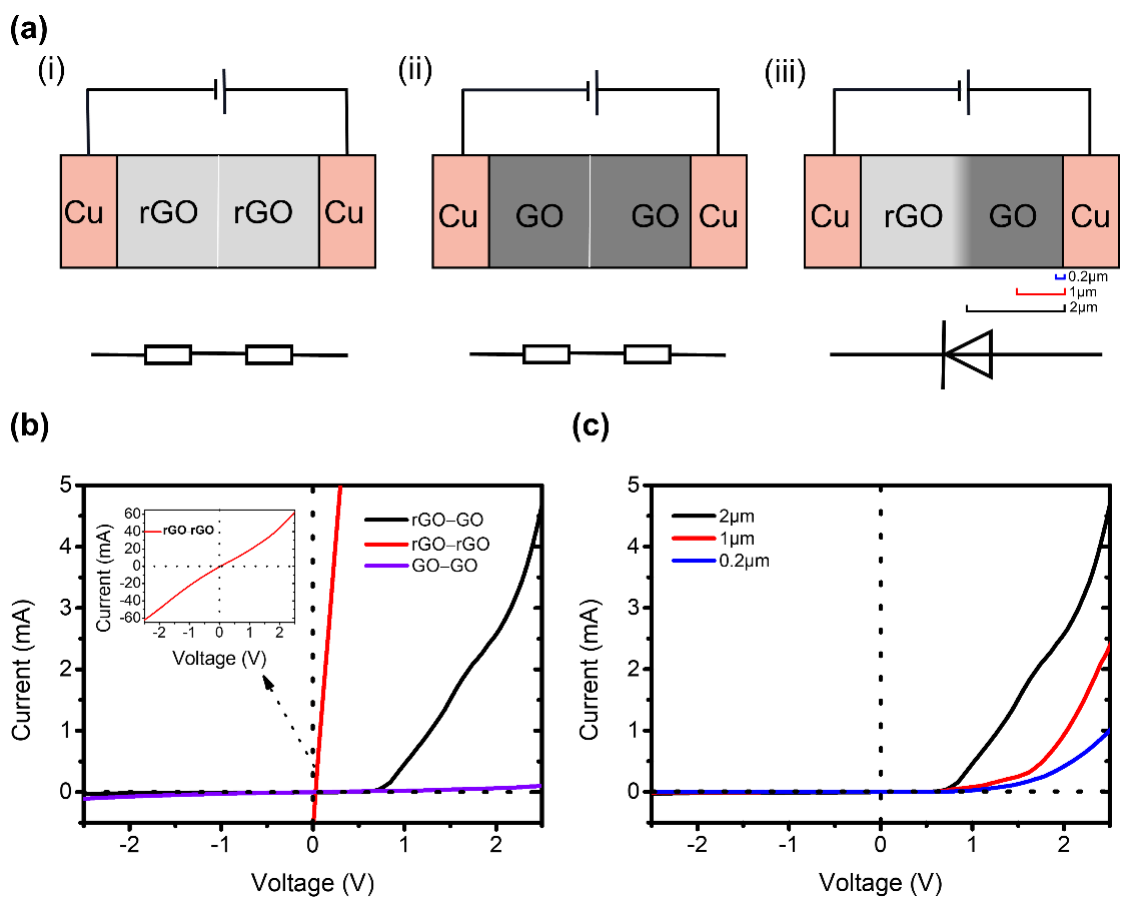
(**a**) Measurement schematic of rGO–rGO, GO–GO films and pGO vertical junctions. (**b**) I–V curves of rGO–rGO, GO–GO films, and pGO vertical junctions. (**c**) I–V curves of pGO vertical junctions with different thickness.

Figure 2a shows the schematic diagram of I–V curves measurement of rGO–rGO, GO–GO and pGO vertical junction devices, respectively. Two copper sheets are used as electrodes to clamp the pGO vertical junctions tightly. The GO film is connected to the anode while the rGO film is connected to the cathode.

The I-V characteristics of rGO–rGO, GO–GO films, and pGO vertical junctions measured are shown in Figure 2b. For rGO–rGO and GO–GO films, the curves show obviously linear relationship, indicating no rectifying behaviors. Interestingly, the I–V curve of pGO vertical junctions shows a great increase with the whole bias voltage range. The current increasing under forward bias is much stronger than that under reverse bias, showing non-linear characteristics and asymmetry of GO–GO film with voltage variation. The current is close to zero at a reverse bias while reaches up to mA level at forward bias, showing a superior rectification performance compared to other lateral graphene p–n junctions, as their μA level of current at the same forward bias [25,26,27]. Moreover, the rectification characteristics of pGO vertical junctions are greater than that of the previous graphene vertical p-n junctions, as summarized in Table 1.

The thickness of the pGO vertical p–n junctions affects the rectification performance. The thickness of rGO remains ~2 μm, and GO films with different thickness were prepared by controlling the amount of GO suspension loaded on the rGO films. We prepared pGO vertical p–n junctions with GO film thickness of ~0.2 μm, 1 μm and ~2 μm, respectively (see the Methods section). As shown in Figure 2c, the forward bias is 2.5 V, and the current of the three GO films with different thicknesses is 1 mA, 2.4 mA, and 4.8 mA respectively. The results indicate that the rectification characteristics of pGO vertical p–n junction increase with the thickness.

**Figure 3:**
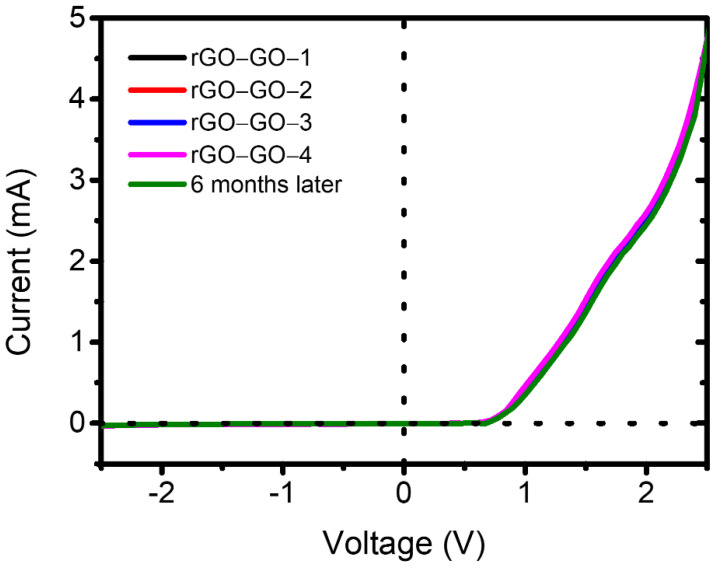
I–V curves of pGO vertical p–n junctions measured multiple times and after six months.

In order to study the stability of rectification effect for our pGO vertical p–n junctions, we have performed multiple measurements on these p–n junctions under ambient conditions. A stable rectification effect was presented after multiple measurements. Moreover, these p-n junctions were sealed and stored in a PE bag for six months, and the p-n junctions showed stability of rectification characteristic over this period of long-term storage (as shown in Figure 3).

**Figure 4:**
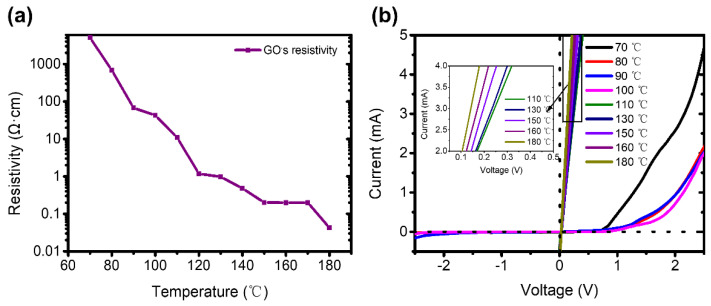
(**a**) Resistivity of GO films with reduction temperature. (**b**) I–V curves of pGO vertical p–n junctions prepared with different reduction temperature.

In addition, we analyzed the effect of reduction temperature of GO on rectification performance. The prepared GO films and pGO vertical p–n junctions were reduced with a corresponding temperature range from 70 °C to 180 °C, respectively (for details, see Method section). Figure 4a shows the resistivity curves of GO films under different reduction temperatures. The resistivity of GO decreases with the increase in reduction temperature, due to the oxygen content of GO decreasing with the increase of reduction temperature. Therefore, the resistivity of GO can be easily and precisely controlled by the reduction temperature of GO. 

As the reduction temperatures affect the resistivity and oxygen content of GO, we measured the I–V curves of pGO vertical junctions reduced at temperature ranges from 70 °C to 180 °C in order to further illustrate the relationship between GO oxygen content and rectification performance of pGO vertical p–n junctions. As shown in Figure 4b, when the reduction temperature of GO is 70~100 °C, the rectification performance of the pGO vertical p-n junctions is obvious. However, with the increase of the reduction temperature, the rectification performance of the pGO vertical p–n junction disappeared when the GO reduction temperature is 110~180 °C. We noted that 110 °C is a critical temperature for the reduction of GO, leading to a sharp reduction in oxygen content of GO, which is consistent with our previous report [34]. This proves that the resistivity or oxygen content of GO affect the rectification performance of the pGO vertical p–n junctions. This is due to the fact that the individual oxygen functional groups present on GO are determined by the reduction temperatures. We found that the predominance of electron–withdrawing groups (i.e., carboxyl, carbonyl, and sp^3^–bonded hydroxyl, ether, and epoxide groups) resulted in p–type GO, while that of electron–donating groups (sp^2^–bonded hydroxyl, ether, and epoxide groups) lead to n-type rGO.

**Figure 5:**
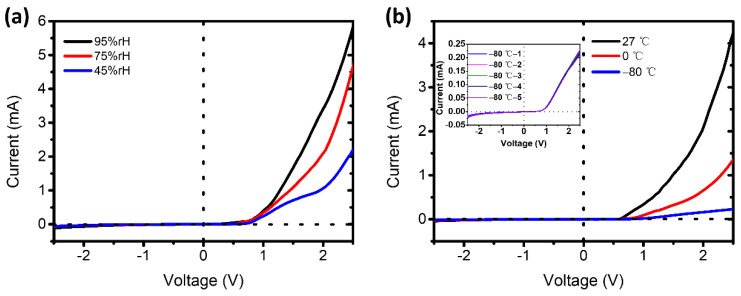
(**a**) I–V curves of the pGO vertical p–n junctions under different humidity. (**b**) I–V curves of the pGO vertical p–n junctions treated with different temperatures.

Then, we further tested the I–V curve of pGO vertical junctions under different humidities (45%, 75%, and 95%) and temperatures (–80 °C, 0 °C, and 27 °C). As shown in Figure 5, the I–V curves of the pGO vertical p–n junctions were sensitive to the humidity and the temperature. There is clear order for the rectification effect for the p–n junctions at different humidities and temperature, which we attribute to the effect of temperature and humidity on oxygen–containing groups in the GO films, as well as the interaction between graphene sheets and the ambient molecules. This demonstrates its potential in a variety of sensing applications, including humidity and temperature sensors [40,41].

### 3.3. Measurement of Hall Effect

To determine the semiconductor type of rGO films, the resistivity, carrier concentration, and mobility of rGO were measured by the Hall measurement system (ET–9000) under ambient conditions. First, the Hall measurement system was pre–calibrated with standard indium–tin oxide (ITO) thin films. Then, rGO films were placed on silicon dioxide substrates for measurement. In addition, the values of each sample were averaged with at least five measurements to ensure reliability. The obtained resistivity, carrier concentration, mobility, and semiconductor type of rGO under ambient conditions were listed in Table 2. Hall effect test results show that the rGO film is a n–type semiconductor, while GO is generally recognized as a p–type semiconductor [42,43], proving the p–n junction performance in our rGO–GO composite films.

### 3.4. Photocurrent Measurement of pGO Vertical p–n Junctions

**Figure 6:**
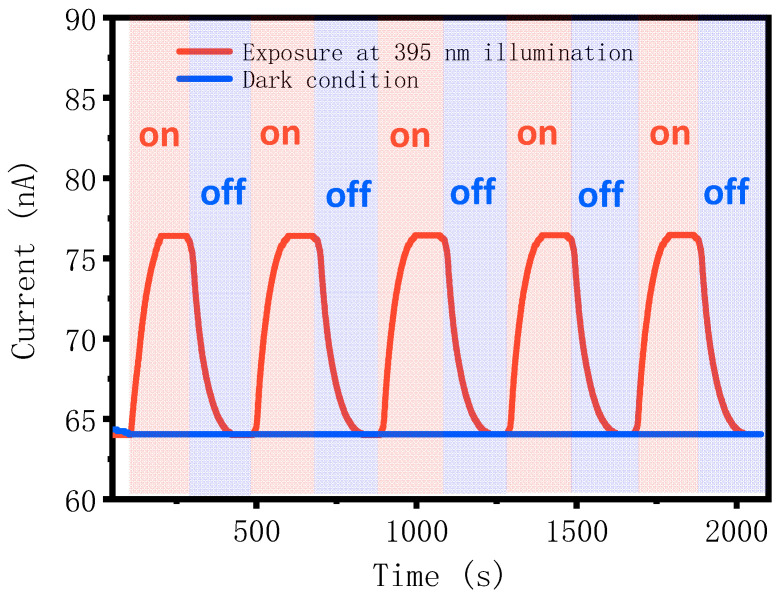
PGO vertical p–n junction photoelectric response.

We measured the photoelectric response of the pGO vertical p–n junction using ITO electrodes under dark conditions and 395 nm laser illumination. After the p–n junction reaches a stable state, it will be exposed to 395 nm laser illumination every 200 s under zero bias voltage. Figure 6 shows the p–n junction generates stable photocurrent of 76.5 nA under 395 nm laser illumination and zero bias voltage. This also proves the formation of p–n junction between rGO and GO films.

### 3.5. Rectification Effect with Different Electrodes

**Figure 7:**
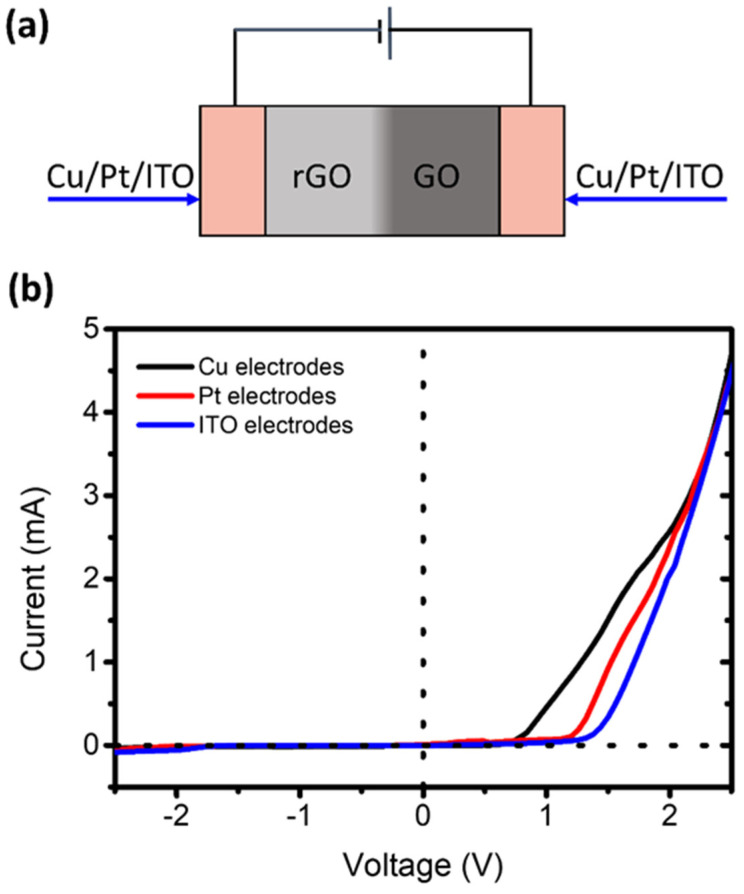
(**a**) Schematic of the pGO vertical p–n junctions measured with copper electrodes, Pt electrodes, and ITO electrodes, respectively. (**b**) I–V curves of the pGO vertical p–n junctions measured with copper electrodes, Pt electrodes, and ITO electrodes, respectively.

We performed experiments to explore the nature of the rectification effect of the pGO vertical p–n junctions. First, we measured the I–V curves of the pGO vertical p–n junctions with copper and ITO electrodes, respectively. The work function of Cu and ITO at room temperature are 4.65 eV and 4.8 eV respectively. Figure 7a shows the schematic of the pGO vertical p–n junctions with different electrodes. As shown in Figure 7b, the I–V curves of the pGO vertical p–n junctions with copper and ITO electrodes showed significant rectification characteristics. Then, a platinum electrode with a larger work function (approximately 5.71 eV) was used to measure the composite film. The I–V curves of the pGO vertical p–n junctions with platinum electrodes also exhibit significant rectification characteristics. Considering the linear curve of pure GO and rGO in Figure 2b, our composite films were demonstrated to be p–n junctions. However, the slight difference between the I–V curves measured by the copper electrodes, the platinum electrodes, and the ITO electrodes indicates that the electrode interacts with GO films may have some influence on the rectification effect.

### 3.6. Theoretical Calculations

**Figure 8:**
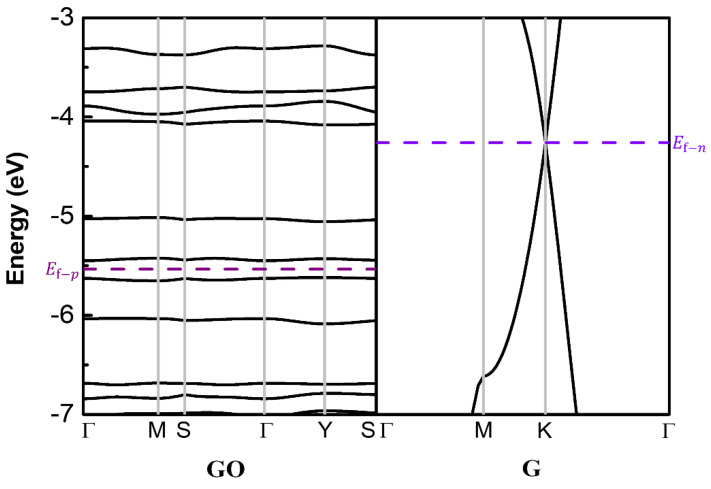
Projected band structure of the model of GO and graphene.

We further performed quantum chemical calculations to illustrate the underlying physical mechanism, using graphene and graphene oxide sheets as examples. As shown in Figure 8, the projected electronic band structure of the GO sheet shows that GO contained 20% oxygen–containing groups has a band gap of ~0.25 eV, indicating a semiconducting property of the GO sheet, while the rGO sheet is considered as an n–type semiconducting in Section 3.3. When the two sheets are combined to form a p–n junction, electrons may flow from the n–zone with high Fermi level to the p-zone with low Fermi level (and vice versa for holes).

## 4. Conclusions

We successfully prepared an undoped GO vertical p–n junction using a two–step drop-casting rGO and GO composite films. The XPS results demonstrated that there are no observable impurity elements signals, proving that the p–n junction is undoped. Remarkably, the prepared pGO vertical p–n junction shows asymmetric rectification behavior, in which the current is close to zero at reverse bias and reaches up to an mA level at positive bias. This rectification performance is superior to those lateral p–n junctions reported previously (since their μA level of current is at the same forward bias). In addition, the p–n junction shows stability of rectification characteristic over long–term storage for six months when sealed and stored in a PE bag, and its rectification characteristic still remain after multiple measurements. This composite film produced an obvious photocurrent at zero bias voltage, indicating the p–n junction characteristics. The junction shows varied rectification effects with thicknesses and oxygen contents of GO, humidity and temperature, showing potential for a variety of sensing applications. Our preparation method has a simple process and is low cost, and the undoped GO vertical p–n junction is not affected by the Klein tunnel effect. Our results demonstrate exciting potential for the enterprise–level production of all–graphene p–n junctions and the creation of all–graphene electrical and optical devices for transparent and flexible electronics and photonics.

## Figures and Tables

**Table 1:** Comparison of figures in graphene p-n junctions.

Material	Type	Doping and Elements	Bias	CURRENT LEVEL	Rectification Ratio	Substrate	Ref.
GO/rGO	vertical	- -	2 V	μA	12:1	- -	[16]
Multi-layer graphene	lateral	Plasma/Nitrogen	5 V	μA	- -	- -	[25]
Graphene/MoS2	lateral	- -	60 V	μA	1.85:1	- -	[26]
Monolayer graphene	lateral	Nanoscale bipolar doping/anions	1 V	μA	2.43:1	SiO_2_/Si (p-doped)	[27]
Graphene/β-Ga2O3	vertical	- -	8 V	μA	8:1	p-GaN	[37]
Monolayer graphene	lateral	Local electrical stress-induced doping	50 V	- -	- -	SiO_2_/Si	[38]
GO(+)/GO(−)	vertical	Chemical functionalization/Tetramethylammonium, N-	1 V	nA	6:1	P+ Si	[39]
GO/rGO	vertical	- -	2.5 V	mA	1459:1	- -	This work

**Table 2:** Resistivity, concentration, mobility, and semiconductor type of rGO films.

Sample	Resistivity (Ω·cm)	Concentration (cm^–3^)	Mobility (cm^2^·V^–1^·s^–1^)	Semiconductor Type
rGO	4.23 × 10^−2^	2.94 × 10^18^	4.44 × 10^1^	N

## Data Availability

Not applicable.

## Sample Availability

Samples of the compounds are not available from the authors upon request.
